# Effect of aquatic resistance interval training and dietary education program on physical and psychological health in older women: Randomized controlled trial

**DOI:** 10.3389/fnut.2022.980788

**Published:** 2022-11-03

**Authors:** Alejandro Martínez-Rodríguez, Bernardo J. Cuestas-Calero, José Manuel García de Frutos, Rodrigo Yáñez-Sepúlveda, Pablo Jorge Marcos-Pardo

**Affiliations:** ^1^Department of Analytical Chemistry, Nutrition, and Food Science, Faculty of Sciences, University of Alicante, Alicante, Spain; ^2^Alicante Institute for Health and Biomedical Research (ISABIAL Foundation), Alicante, Spain; ^3^Faculty of Sport, San Antonio Catholic University of Murcia, Murcia, Spain; ^4^Faculty of Education and Social Sciences, Universidad Andres Bello, Santiago, Chile; ^5^Department of Education, Faculty of Education Sciences, University of Almería, Almería, Spain; ^6^SPORT Research Group (CTS-1024), CERNEP Research Center, University of Almería, Almería, Spain

**Keywords:** body composition, strength, geriatric rehabilitation, ageing, aquatic resistance training, physical performance, older adults

## Abstract

Due to demographic changes, the world’s population is progressively aging. The physiological deterioration of the older adult may lead to reduced balance capacity and increased risk of falls, among others, due to the prevalence of degenerative diseases. Physical exercise can be effective in reducing the risk of disease and slowing functional decline in older people. The aim of the research is to test the effects of aquatic resistance training and dietary education on health indicators, strength, balance, functional autonomy, perception of satisfaction with life. Thirty-four participants aged 69 ± 4 years were randomly assigned into two groups: experimental (aquatic resistance interval training) and control group (no intervention). The intervention consisted of resistance training in an aquatic environment carried out for 14 weeks (three sessions per week: 60 min each). All variables were analyzed twice; pre - post intervention. Aquatic resistance training has positive effects on strength (*p* < 0.001), functional self-sufficiency (*p* < 0.001) and aerobic capacity (*p* < 0.001), however, no significant differences were observed in the perception of satisfaction with life and balance. Research results suggest that older women who engage in regular, scheduled aquatic resistance training have greater autonomy in performing activities of daily living, agility, gait control, and body composition variables (lower fat compartment and greater muscle mass).

## Introduction

Aging is linked to a functional deterioration in human beings and can become a serious problem due to its prevalence of degenerative diseases (1). Therefore, physical exercise can be effective to reduce the risk of disease and slow functional deterioration in the older people (2–4). An aspect of extraordinary importance in adults and the older people is the decline in functional capacity with advancing age, a predictable phenomenon that can be halted or slowed down by paying special attention to the level of physical fitness (body composition, strength, endurance, flexibility, etc.) and physical activity.

This functional decline can affect the physical, cognitive, and psychological functions of older adults, as well as life satisfaction. In a observational study carried out between 1994 and 2014 (5) they observed that the effect of physical health on later mental health is greater than the effect of mental health on later physical health in later life. In addition, a clear positive association between psychological well-being and short- and long-term health outcomes and quality of life has been found (6). Many adults and older people, due to their sedentary lifestyles, are dangerously close to their maximum capacity level, just performing normal activities of daily living (7). A small decrease in the level of physical activity in these individuals could result in the transition from a state of independence to a state of disability, which will be characterized by the need for assistance in performing co-occurring activities of daily living. Therefore, prevention of dependence takes on a special dimension in order to avoid the deterioration of quality of life and dependence in the older people (8).

As age increases, moreover, the rate of falls can increase by as much as 60% (1, 9). Three of the most common modifiable intrinsic (subject-related) fall risk factors are muscle weakness (relative risk ratio/odds ratio 4.4), balance deficit (relative risk ratio/odds ratio 2.9) and gait instability (relative risk ratio/odds ratio 2.9) (10–14). In addition to balance, muscle strength/power is required (1). The general causes of age-related skeletal muscle mass loss (i.e., sarcopenia) are multiple (e.g., cellular, neural, metabolic, and hormonal contributors).

Physical exercise is considered one of the most important factors in improving quality of life in older people, due to improved functional capacity, decreased risk of falls, and improved gait ability, balance, cardiorespiratory capacity, and muscle strength development (15–18). Despite the numerous benefits, regular exercise is difficult to achieve for many older adults, as participation levels often decline with age (19). Decreased participation levels vary due to a myriad of factors such as type of physical activity, age, health problems, pain, and perception of disability (19). As per recommendation by the American College of Sports Medicine and the American Heart Association, water-based exercise is a safe and useful alternative to land-based exercise for older adults or individuals with limited tolerance for weight-bearing activities (20).

In particular, strength exercise in an aquatic environment has been related to improved balance (21–24), as the buoyancy force of the water and hydrostatic pressure/density help participants to slow down movement, and the additional sensory cues provided by the viscosity of the water facilitate the timing of muscle recruitment (25). Thus, aquatic exercise can ameliorate the negative physiological effects of aging, which are modifiable risk factors and predisposing factors for fall (26). On the other hand, interval training has been related as an alternative method for health improvement (27), so it could be related to increased performance and improvement of blood pressure, lipid profile, improved metabolic condition and strength gain (28, 29). Also, it should be noted that there is an indirect relationship between fitness factors and some components of body composition, such as fat mass (30). In this regard, the importance of adequate nutritional education should also be emphasized, since sufficient protein intake has been shown to counteract the effects of sarcopenia in older adults, noting that, although energy requirements are lower in old age, the requirements for many other nutrients may not change or may even increase. Changes in the balance of nutrient-rich versus less nutrient-dense foods have been shown to occur, contributing to lower protein and micronutrient intakes (31).

To date, there is no research that studies the effect of interval resistance training in an aquatic environment together with nutritional education on both physical and psychological variables in older women. In this context, a water-based interval resistance training intervention was chosen as a potential candidate to provide improvements in functional capacity, balance, strength, body composition, flexibility, aerobic capacity, and satisfaction with life in women over 65 years of age. For that, the aim of the research was to find out the effects of aquatic resistance training and dietary education on physical and psychological health in older women.

## Materials and methods

### Study design

In this randomized controlled trial study, participants were assigned to an experimental group (EG = aquatic resistance interval training and nutritional education) and control group (CG = only nutritional education) to determine the efficacy of aquatic resistance interval training on the variables of on the variables of strength, functional autonomy, body composition, static standing balance, flexibility, aerobic endurance, and satisfaction with life. Allocation was electronically randomized by two-arm block design using online computer software, as indicated by published recommendations (32). An investigator who was not involved in the interventions or assessments in this study performed this procedure. Previously, sports centers in the province were contacted and an invitation to participate in the research was sent out. An informative talk was given in which they were informed of the objective and their collaboration was requested.

### Participants

**FIGURE 1:**
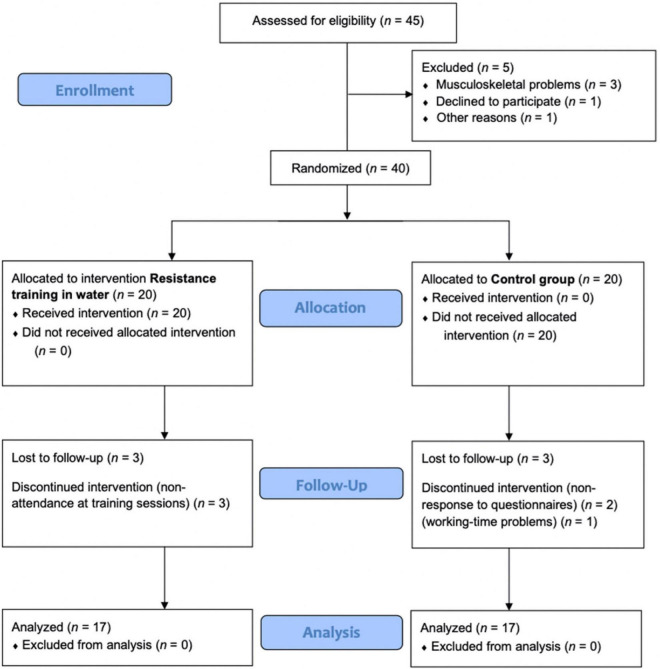
Consort 2010 flow diagram.

Only female older adults participated in the investigation. Thirty-four women chose to participate; 17 were in the experimental group (69.4 ± 4.9 years old) and 17 in the control group (67.7 ± 3.6 years old). Being over 65 years of age; not having undergone surgery in the last year; not having musculoskeletal, neurological, or orthopedic diseases that could affect the ability to perform the tests and being able to walk independently without orthopedic assistance were the established inclusion criteria. After the initial assessment, one participant refused to take part in the research and four others had mobility problems. Forty women were randomly assigned, however, during the intervention six women withdrew from the study for personal reasons. Thirty-four women were included in the analysis. This procedure was established according to “CONSORT” statement^1^, as displayed in the flowchart in Figure 1.

### Declarations: Ethical approval, consent to participate, and consent for publication

The norms of the Declaration of Helsinki were considered in the development of the research. The University Human Research Ethics Committee of Alicante University (Spain) appoved the research; code UA- 2018-10-22. This trial was registered at clinicaltrials.gov as NCT05052164^2^. All participants signed an informed consent form after being informed of the benefits, risk, and detailed description of this research. All data were coded to maintain the confidentiality of the study participants.

### Intervention

The water exercise was conducted and supervised by master in sport science specialist also in accordance with physical exercise prescription for older adults established by the American College of Sports Medicine (33).

The participants completed 14 weeks of water exercise training program three times a week (42 sessions), with 60 mins per session which was comprised by 15 mins of warm-up (10 mins of aerobic and resistance exercises and 5 mins of stretching), 30 mins of water interval resistance training (Table 1), followed by 15 mins of cool down (5 mins of stretching and 10 of relaxation exercises). This intervallic training consisted of four sets of 5 min of training with 2 min rest between sets. During the 5 mins of exercise there was no rest. In each session, the same exercises (pectoral/back, hip flexor/extensor, biceps/triceps, knee flexor/extensor, shoulder, and core) were performed for 1 min consecutively, with intervals of 30, 20, and 10 s, and at low, moderate and high perceived intensity, respectively. Finally, relaxation exercises (10 min) and stretching of all muscle groups (5 min) were performed. All women in the experimental group participated in each of the sessions. The Borg scale was used in each of the sessions to measure the perception of effort (34), as well as to indicate whether they should perform the exercises at low, moderate or high intensity. After each training session, they were asked about their fatigue state. To meet 85% adherence the subjects were informed that they could not miss more than six sessions. Session attendance was recorded by the sport science specialist.

**TABLE 1:** Aquatic resistance interval training.

Weeks	Volume × Intensity Hate-rate × sec	Total, time Total, exercise
1–4	4 × [5 min (30 sec 30%; 20 sec 60%; 10 sec 90%) + 2 min rest] 30 sec (45.6 ± 4.94); 20 sec (91.2 ± 4.94); 10 sec (136.8 ± 4.94)	30 min 20
5–14	4 × [5 min (30 sec 30%; 20 sec 60%; 10 sec 90–100%) + 2 min rest] 30 sec (45.6 ± 4.94); 20 sec (91.2 ± 4.94); 10 sec (136.8/152 ± 4.94)	30 min 20

%, heart rate; min, minutes; sec, seconds; rest, recovery between sets.

Physical activity was measured by means of the IPAQ questionnaire (35) at the beginning and end of the study. Complementarily, all participants received the same nutritional education, based on the Mediterranean diet, divided into four 60-min theoretical-practical workshops over 14 weeks, with the aim of providing updated information on the benefits of following a proper dietary pattern. Trained dietitians led the sessions. In addition, these dietitians were in contact with the sport science specialist, who led the training sessions. In this way, if any questions arose, they were resolved in these workshops. The PREDIMED Mediterranean diet adherence questionnaire was used to control dietary habits of the participants. Participation was 100%, i.e., all patients attended all workshops. These workshops were conducted to prevent eating habits from being a potential confounding factor in the results obtained. Both the water resistance training intervention and the nutritional workshops took place at the Catholic University of Murcia, during the last months of 2020 and early 2021.

### Outcome measurements

#### Body composition

The weight and height of all participants were measured with high-quality calibrated electronic scales and a mobile anthropometer (Seca 213, SECA Deutschland, Hamburg, Germany), respectively. The participants were dressed in light clothing and without shoes. Using weight in kilograms and height in centimeters, body mass index (BMI) was calculated as weight/height^2^ (kg/m^2^). Body perimeters were measured in triplicate (with subsequent averaging) with an anthropometric tape. Skinfolds (subscapular, tricipital, bicipital, iliac crest, supraspinal, abdominal, anterior thigh, and middle leg) were obtained with a Holtain Skinfold Caliper. Three bone diameters were also measured, using a small bone diameter pachymeter (Smartmet, Jalisco, Mexico; accuracy, 1 mm). The mean technical error for, circumferences, lengths and heights was less than 1% and for skinfolds less than 5%. All these measurements were performed by anthropometrists accredited by the International Society for the Advancement of Kinanthropometry (ISAK) level 2, according to ISAK guidelines (36). The sum of 6 folds was calculated from the tricipital, subscapular, supraspinal, abdominal, thigh, and leg folds. The rest of the measurements were used to calculate muscle mass, based on the five-component model proposed by Kerry Ross (37).

#### Functional autonomy

The protocol of the Group of Latin-American Development for Maturity (GDLAM) was used to evaluate functional autonomy (38) in its validated version for the Spanish population (28). It defines functional autonomy covering three aspects: autonomy of action that relates to the notion of physical independence; autonomy refers to the possibility of self-determination and autonomy than allows the person to judge any situation. GDLAM protocol is composed by the following five tests:

(1) 10 m walk (W10 m); the purpose of this test is to evaluate the individual’s speed to travel 10 m.

(2) Getting up from a seated position (GSP); the test is intended to evaluate the functional capacity of the lower extremity. The individual, starting from a sitting position on a chair, without the support of the arms, with the seat at 50 cm from the floor, stands up and sits down five consecutive times.

(3) Getting up from the prone position (GPP); the purpose of this test is to evaluate the individual’s ability to get up from the floor. Starting from the initial position of ventral decubitus, with the arms along the body, after giving the command, the subject must get up and stand up as quickly as possible.

(4) Getting up from a chair and movement around the house (GCMH); the objective is to evaluate the individual’s ability, in relation to agility and balance, in common day-to-day situations. With a fixed chair on the floor, two cones should be placed diagonally and behind the chair, at 4 m to the back and to the right and left sides of the chair. The individual begins the test sitting on the chair, with feet raised off the floor and, at the observer’s command, stands up, goes to the right, circles the cone, returns to the chair, sits down and lifts both feet off the floor. Without hardly resting, he does the same movement to the left.

(5) To put on and take off a T-shirt (PTS): the individual should be standing with arms along the body and a T-shirt in one of the hands. At the voice signal of “Go,” the individual should put on the shirt and immediately take it off, returning to the starting position. This test is intended to measure the agility and coordination of the upper limb.

All tests were individually conducted and repeated two different times with a minimum of 5 min intervals, the lowest time of the two trials was recorded. After performing this battery of tests, the GDLAM index (GI) is calculated, where the lower the value of the score, the better the result, using the following formula: GI = [(W10 m + GSP + GPP + PTS) × 2] + GCMH]/4. All the tests were measured using time in seconds. It classifies the subjects as: weak GDLAM, with figures of >28.54; fair, between 28.54 and 25.25; good, between 25.24 and 22.18, and very good, below 22.18 points.

#### Maximal isometric hand grip force test

The manual grip strength of the upper limbs was measured by means of a dynamometer adjusted for each type of hand, and on a scale from 0 to 100 kg. The individual was in the orthostatic (standing and upright) position with the arm to the side of the body and with the dominant side performed the grip (39). The participants performed one repetition in each hand to familiarize themselves with the device and the test. Each participant was asked to squeeze the grip with maximal strength for 3 s with the dominant hand. The highest peak strength (kg) recorded between the three attempts was considered for analysis. A digital grip strength dynamometer was used for this (TKK 5401; Takei Scientific Instruments Co., Ltd., Tokyo, Japan). Dividing the value of the best hand grip strength score by BMI (kg/m^2^) obtains a field muscle quality index (MQI) (40).

#### Isometric strength

Isometric strength of the quadriceps was performed with the load cell maximal isometric strength test (41, 42). To determine the maximal isometric knee extension strength, the participants were assessed while seated with a knee and hip angle of 90°. Participants seated in a knee extension machine were instructed to push as strong as possible for 3 s while provided with verbal encouragement. The extension test was assessed with a load cell force transducer (Musclelab, Ergotest, Norway) sampling at 1,000 Hz. The subjects performed three IKE tests with 2 min of rest between tests. The maximum peak force in Newton (Nw) was collected.

#### Postural stability tests

The static standing balance test aims to maintain the verticality of the body in static situations. The way to evaluate balance in older people is the one proposed by Onambele et al. (43). Three types of balance were performed: (1) bipedal stance, (2) single-leg stance, and (3) tandem stance, all of them with eyes open. A force platform (MuscleLab force plate, 200 Hz/1 kHz, Ergotest Technology a.s., Stathelle, Norway) was used, acting as a switch as they are very useful to record contact times between supports. Subjects were barefoot throughout the whole exercise and were asked to stand quietly with hands hanging freely at either side, looking straight ahead at a target (black circle 15 cm in diameter against a white background) placed at eye level, ∼3 m away.

#### Aerobic endurance

The 6-min walk test (44) was used. The women walked (without running) the longest possible distance for 6 mins, in a 45.72 m course marked in segments. It was performed in an enclosed, well-lit room on a non-slip surface. The women who needed it stopped to rest and resumed the test. The evaluator warned when there were 3-, 2-, and 1-mins left. The result was recorded as total meters walked.

#### Flexibility

The Chair Sit and Reach flexibility test (44) in its adapted and validated version for older adults (45) was used to measure flexibility. A 45 cm high (17 inch) chair with a backrest and attached to the wall to prevent it from moving was used, as well as a measuring tape. Participants sat on the edge of the chair resting one foot on the floor with the leg flexed at hip width and the other leg straight with the foot in 90° dorsal flexion. They stretched their arms in front of the straight leg with one hand on top of the other and palms down, trying to touch or overlap the tip of their toes with the middle finger, maintaining the maximum trunk flexion position for 2 s, keeping the spine as straight as possible and the head in normal alignment with the spine (not cramped).

#### Satisfaction with life

The satisfaction with life scale (SWLS) is a 5-item scale that assesses life satisfaction (46). The responses are classified in a 7-point Likert scale. This scale has been found to have favorable psychometric properties, including high internal consistency and reliability, and has been consistently used to measure life satisfaction in several countries (47, 48).

### Statistical analyses

Jamovi 1.1.3.0 software was used to perform all statistical analyses. Descriptive statistics (mean ± standard deviation) were calculated for all the variables and the normality distribution was tested using the Shapiro-Wilk test. For equality of variances, Levene’s test was performed, and analysis of covariance (ANCOVA) was applied (general linear model; time × group) with BMI as a covariate to analyze the effects of the intervention on the assessments. For time × group interaction effects, omega squared effect sizes were calculated. If significant main effects were found, *post hoc* (Bonferroni) tests were performed. Moreover, to set up connections between the variables of the study, the Pearson’s correlation test was used in the correlations to determine the effect size (small: 0.10, medium: 0.30 and high: 0.50) (49, 50), with 95% confidence intervals. The level of statistical significance was set at *p* ≤ 0.05.

## Results

The baseline data of the sample are presented in Table 2. Statistically significant differences were observed between the experimental (water) group and the control group in terms of height and weight, being greater in both cases in the water group. Regarding the possible confounding variables, physical activity and adherence to the Mediterranean diet, no differences were observed in the control group for physical activity. In the experimental group there was an increase due to the intervention. Adherence to the Mediterranean diet did not change significantly in the experimental group (5.7 ± 2.0 vs. 5.9 ± 2.36; *p* = 1.000) and in the control group (6.1 ± 2.1 vs. 5.5 ± 2.3; *p* = 0.75).

**TABLE 2:** Baseline characteristics of study participants.

	Experimental		Control	
	Mean	SD	Mean	SD
Height (cm)	161*	7.95	154*	5.47
Weight (kg)	75.4*	12.4	66.9*	10.2
Age (years)	69.6	5.01	67.7	3.60

cm, centimeters; kg, kilograms; SD, standard deviation. *Mean differences were significant at *p* < 0.05.

### Body composition

Table 3 show the body composition variables from EG and CG, respectively, at the beginning or end of the study for women. Statistically significant differences were observed in the summary of folds and muscle mass variables. For the sum of folds, a decrease in the sum of folds was observed in the experimental group (*p* < 0.001) and an increase in the control group (*p* < 0.001). In addition, there are significant differences between both groups after the intervention (*p* = 0.014). Muscle mass increases significantly in the experimental group (*p* < 0.001) and after the intervention they present significantly higher values than women in the control group (*p* = 0.016). No significant differences were observed for waist, hip and thigh circumferences.

**TABLE 3:** Body composition variables on pre- and post-training moments of the resistance training and control groups.

	Experimental group				Control group				Effect time			Effect time × Group		
	Baseline		Post		Baseline		Post							
						
	Mean	SD	Mean	SD	Mean	SD	Mean	SD	*F*	*p*	ω^2^	*F*	*p*	ω^2^
Waist (cm)	88.5	11.5	88.3	11.5	89.6	8.91	88.7	10.2	1.256	0.270	0.006	0.646	0.427	–0.008
Hip (cm)	104	11.4	104	11.7	103	6.48	104	7.59	0.207	0.652	–0.002	2.126	0.154	0.027
Thigh (cm)	49.3	5.68	50.4	5.64	49.2	5.03	49.2	5.48	2.52	0.122	0.036	2.18	0.149	0.028
Σ 6 skinfolds	128	39.7	113	35.8	140	29.7	151	31.6	1.86	0.182	0.021	25.36	<0.001	0.375
Muscular mass (Kg)	30.4	5.41	32.5	6.1	27.8	4.71	26.6	4.45	1.36	0.253	0.008	60.05	<0.001	0.593
Muscle/bone index	2.83	0.373	3.24	1.09	2.79	0.359	2.68	0.374	1.53	0.225	0.013	4.77	0.036	0.085

cm, centimeters; kg, kilograms; SD, standard deviation; ω^2^, omega squared.

### Functional autonomy

For the level of functional autonomy, both groups presented fair to good functional autonomy according to the reference values (Table 4). After the intervention, it was observed that the experimental group improved its functional capacity significantly (*p* < 0.001). Furthermore, after the intervention, significantly lower values were observed in the experimental group (*p* = 0.001) and therefore a higher degree of functional autonomy. Considering that the GI was designed to represent the degree of functional autonomy in the older people, and that healthy aging depends on the level of functional status, it seems that physical exercise increases this capacity.

**TABLE 4:** Functional capacity and muscular strength variables on pre- and post-training moments of the resistance training and control groups.

	Experimental group				Control group				Effect time			Effect time × Group		
	Baseline		Post		Baseline		Post							
						
	Mean	SD	Mean	SD	Mean	SD	Mean	SD	*F*	*p*	ω^2^	*F*	*p*	ω^2^
**GDLAM**														
10 mW (sec)	6.25	1.15	5.59	1.06	7.26	1.35	7.39	1.17	6.34	0.017	0.116	13.88	<0.001	0.241
GSP (sec)	8.95	1.69	8.77	1.78	12.1	2.64	12.8	2.57	1.17	0.287	0.041	3.45	0.072	0.057
GPP (sec)	3.71	1.02	3.62	0.984	6.14	2.70	6.08	2.97	0.20	0.655	–0.020	0.01	0.912	–0.024
GCMH (sec)	51.9	9.67	41.3	5.15	56.3	7.45	53.3	8.06	58.5	<0.001	0.586	17.8	<0.001	0.293
PTS (sec)	12.2	3.38	11.2	3.21	14.5	5.00	15.6	6.29	9.61	0.975	0.175	4.66	0.038	0.083
GI GDLAM	28.6	3.97	24.7	2.75	34.1	6.40	34.2	7.27	23.0	<0.001	0.352	25.4	<0.001	0.376
**Handgrip**														
HG D (kg)	25.5	5.96	29.1	7.25	23.4	3.12	22.5	3.75	16.6	<0.001	0.278	40.3	<0.001	0.492
HG ND (kg)	24.2	6.44	26.8	6.97	21.4	3.91	20.8	3.84	16.6	<0.001	0.278	40.3	<0.001	0.492
MQI	0.89	0.26	1.03	0.29	0.84	0.15	0.81	0.16	0.686	0.414	0.007	29.9	<0.001	0.416
**Load cell quadriceps extension**														
Max. force (N)	221	96.7	301	113	202	70.9	178	61.9	16.3	<0.001	0.274	55.2	<0.001	0.572
Time (sec)	4.45	1.73	4.40	1.75	4.53	2.00	4.02	2.14	0.491	0.488	–0.012	0.31	0.578	–0.011

SD, standard deviation; HG, handgrip; D, dominant; ND, no dominant; MQI, muscle quality index [handgrip strength (kg)/BMI (kg/m^2^)]; 10 M, 10 m walk; GSP, to get up from the sitting position; GPP, to get up from the ventral decubitus position; GCMH, getting up from a chair and movement around the house; PTS, put on and take off a T-shirt; sec, seconds; Max, maximum; N, newton; ω^2^, omega squared.

### Isometric strength

For the handgrip test (Table 4), significant differences were observed for both dominant and non-dominant hands, both over time and between groups. In the experimental group, a significant increase was observed in both the dominant (*p* < 0.001) and non-dominant (*p* < 0.001) hands after the intervention. In addition, significant differences were observed after the intervention between groups, with significantly higher values in the experimental group (*p* = 0.011 and *p* = 0.022, dominant and non-dominant hand, respectively). For the muscle quality index (Table 4), significant differences were observed in the experimental group before and after the intervention (*p* < 0.001), with a higher value after the intervention. In addition, the experimental group presented a significantly higher value than the control group at the post-intervention time (*p* = 0.026). Regarding the maximum force in Newton (Nm) measured with a load cell force transducer (Musclelab, Ergotest, Norway), it was observed that there were significant differences in the intervention group after the intervention (*p* < 0.001) and between both groups at the time post (*p* = 0.002).

### Postural stability tests

**FIGURE 2:**
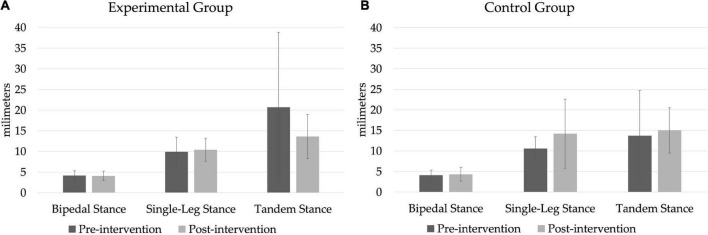
Postural balance tests on a force platform. **(A)** Experimental group, **(B)** control group.

In none of the three postural stability tests (Figures 2A,B) were significant differences found in either group.

### Flexibility and aerobic endurance

**FIGURE 3:**
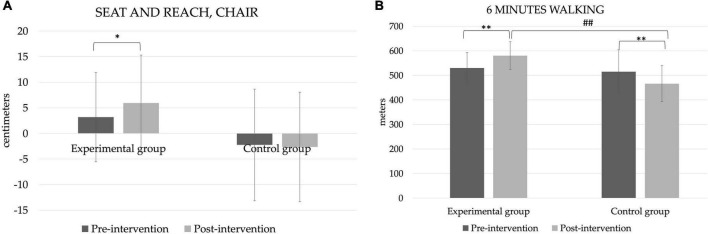
Flexibility and resistance. *p < 0.05 and **p < 0.01 by intragroup analysis. ^##^*p* < 0.01 by intergroup analysis. **(A)** Seat and reach, chair, **(B)** 6 mins walking.

After determining the flexibility of the participants and analysing the results, it was observed that at the initial moment there were no significant differences (Figure 3A). Only in the experimental group there is a significant increase (*p* = 0.003) after the intervention. As for endurance (Figure 3B), prior to the intervention the results were 530 ± 63.9 and 515 ± 89.9 m, for the experimental and control groups, respectively. After the intervention, a significant increase was observed in the experimental group (580 ± 56.7; *p* < 0.001) and a decrease in the control group (466 ± 73.1; *p* < 0.001). There were also differences between the two groups at post moments (*p* < 0.001).

### Satisfaction with life

**FIGURE 4:**
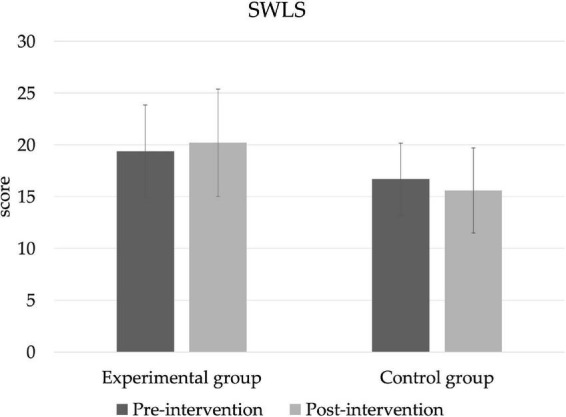
Satisfaction with life (SWLS).

The SWLS scores can be seen in Figure 4. There are no significant differences in scores between groups or at different points in time; however, in the experimental group the score goes up slightly, while in the control group it goes down.

The analysis of the correlations between all participants and each variable are shown in the Table 5. Statistically significant positive correlations are observed between the variables sit and reach and walking 6 mins (*p* = 0.044), as well as with the subscales of the GDLAM, walking 10 m (*p* < 0.001), GSP (*p* = 0.12), GPP (*p* = 0.002), GCMH (*p* < 0.001), and the total index (*p* < 0.001). As for the life satisfaction questionnaire, it is positively related both with the handgrip results (*p* = 0.002) and with the maximum strength result in loading platform (*p* = 0.006) and muscle mass (*p* = 0.010), in a significant way. Finally, in the case of stability, significant correlations were found between the tandem variable with some of the GDLAM subscales and with the GI (*p* = 0.003), as was the case with the single-leg stance variable (*p* = 0.013).

**TABLE 5:** Correlations between variables increments (post-intervention–basal).

	HG D	HG ND	Sit and reach	6 min W	Max. Force (N)	Time (sec)	Bipedal stance	Tandem stance	Single-leg stance	10 mW	GSP	GPP	GCMH	PTS	IG	SWLS	THIGH	Skinfolds	MM
HG D	–																		
HG ND	0.925**	–																	
Sit and reach	0.088	0.149	–																
6 min W	0.562**	0.566**	0.343*	–															
Max. force (N)	0.724**	0.707**	0.098	0.602**	–														
Time (sec)	–0.098	0.013	–0.090	–0.136	–0.017	–													
Bipedal stance	0.091	0.146	0.007	–0.159	–0.139	0.068	–												
Tandem stance	0.013	0.005	–0.275	–0.320	–0.124	0.294	0.439*	–											
Single-leg stance	–0.013	–0.094	−0.528*	−0.386*	0.009	0.109	0.046	0.309	–										
10 mW	−0.486*	−0.501*	−0.590**	−0.686**	−0.492**	0.071	–0.050	0.174	0.418*	–									
GSP	−0.479*	−0.463*	−0.420*	−0.539**	−0.563**	–0.010	0.032	0.311	0.112	0.604**	–								
GPP	−0.445*	−0.481*	−0.509*	−0.659*	−0.408*	0.294	0.141	0.415*	0.541**	0.659**	0.701**	–							
GCMH	−0.504*	−0.542**	−0.539**	−0.760**	−0.541**	–0.027	0.185	0.365*	0.385*	0.777**	0.777**	0.805**	–						
PTS	–0.071	–0.056	−0.492*	−0.448*	–0.217	0.237	0.377*	0.600**	0.428*	0.511*	0.492*	0.663**	0.610**	–					
GDLAM IG	−0.406*	−0.413*	−0.583**	−0.690**	−0.481*	0.130	0.229	0.495*	0.414*	0.759**	0.827**	0.881**	0.917**	0.832**	–				
SWLS	0.516*	0.449*	–0.020	0.326	0.459*	0.172	0.191	–0.047	0.177	−0.395*	−0.393*	–0.151	–0.287	–0.144	–0.296	–			
Thingh	0.113	0.034	0.057	–0.119	–0.070	–0.020	–0.069	–0.026	0.037	0.027	0.043	–0.002	0.057	–0.058	–0.012	0.015	–		
Skinfolds	–0.166	–0.215	–0.033	−0.494*	−0.356*	–0.090	–0.116	0.203	0.074	0.197	0.288	0.236	0.306	0.187	0.280	–0.148	0.547**	–	
MM	0.522*	0.426*	0.062	0.209	0.242	–0.028	0.153	–0.109	–0.004	–0.292	−0.395*	–0.318	–0.293	–0.186	−0.339*	0.427*	0.668**	0.121	–

*P-value < 0.05; **p value < 0.01. HG, handgrip; D, dominant; ND, no dominant; min, minutes; W, walking; N, Newtons; max., maximum, 10 M, 10 m walk; GSP, to get up from the sitting position; GPP, to get up from the ventral decubitus position; GCMH, getting up from a chair and movement around the house; PTS, put on and take off a T-shirt; SWLS, satisfaction with life; MM, muscular mass.

## Discussion

The aim of this study was to analyze the efficacy of the addition of aquatic resistance interval training program and dietary education on body composition, functional capacity, balance, strength, aerobic capacity, flexibility, and satisfaction with life in women over 65 years of age. Performing training in an aquatic environment in older population allows providing a light and low-impact environment where people can exercise safely (26), where buoyancy, pressure, resistance, and water temperature maximize the effectiveness of aquatic exercise, allowing light and safe body movements (51).

The present study reveals several findings: aquatic resistance interval training for 14 weeks improves body composition, upper and lower body strength, increases functional autonomy, muscle mass, flexibility, and aerobic capacity. However, the stability variables showed no statistically significant differences. Regarding body composition, in the training group, significant improvements were observed in the variables of fold sum and muscle mass, coinciding with results already published (23, 52, 53), in which programmed training caused decreases in body fat. Muscle mass has a fundamental role in the older population, since there is evidence that, without intervention, sarcopenia, and frailty often lead to disability, falls and a decrease in quality of life (54).

The results of the present investigation are in agreement with previous findings, where aquatic exercise has been shown to elicit positive responses on FA in the population between 50 and 80 years of age (55). After the intervention, the FA of older people who underwent aquatic resistance interval training improved significantly, with no improvement in CG. In addition, FA was positively correlated with stability values, so it seems that women with greater stability and muscle mass have greater functional autonomy. In this sense, it is confirmed that systematic and controlled training, widely evidences ostensible improvements in older adults, especially in functional autonomy (56).

Stability is considered one of the most important variables when designing a training program for fall prevention. Research has shown that resistance training in an older people population can improve static balance (57, 58), however, not all of them always get these same conclusions (26). In the present study, no significant differences were observed between groups in terms of stability variables. The lack of significant results may be due to the lack of specificity of the training to improve this variable (26).

The properties of water provide unique opportunities for rehabilitation through the hydrostatic and hydrodynamic principles of buoyancy and resistance (59), which consequently contributes to reduced pain, stiffness, and difficulty in physical functions. Consequently, physically active older adults across the lifespan have higher levels in terms of physical and cognitive function, mobility, less musculoskeletal pain, lower risk of falls and fractures, depression, and better quality of life. Therefore, the results systematized by Fuentes et al. (55) are in line with the results obtained after the present investigation.

After the intervention, a significant increase in isometric strength of both upper and lower limbs was observed. Previous findings show that limited mobility during aging is associated with the loss of strength and/or function that characterizes sarcopenia (60). In particular, when training is performed in an aquatic environment, it should be taken into account that the density of water is an important characteristic since it can generate an increase in muscle strength because movement in water offers 900 times greater resistance than in air (61). In the correlations, it is also observed that women who have more upper and lower body strength and greater muscle mass show higher values in the satisfaction with life questionnaire.

In the review conducted by Martinez-Carbonell (26) included six studies that evaluated flexibility as an outcome after aquatic training, in all cases, they observed that flexibility improved significantly from pre-test to post-test. This study confirms these results, as significant improvements were only observed between pre- and post-test in the experimental group. It seems that this may be due to decreased stiffness in the pelvic muscles, improving gait and decreasing the risk of falls (26). It has been shown (26) that in order to observe significant changes in flexibility in older adults, training should last a minimum of 12 weeks, with two to three sessions per week of about 60 mins duration. The intervention performed in the present investigation met the requirements. Finally, after the intervention, the distance walked in 6 mins improved in the experimental group. This corroborates current findings, which show that resistance training improves aerobic capacity (62), as interval resistance training is also effective in improving cardiac, respiratory and metabolic function in an older adult population.

In terms of life satisfaction, improvements were observed, although not significant in the experimental group. Positive effects on life satisfaction have previously been found after both shorter (e.g., 12 weeks) and longer (e.g., 8 months) interventions (63). It appears that these improvements may be since resistance training influences some areas of psychological functioning, in addition to improving physical function, increasing the ability to perform activities of daily living and decreasing pain.

The present study has some limitations that should be considered when interpreting or applying our results. First, the sample is only composed of women. It is unclear whether male participants would receive similar benefits from aquatic training, and more research needs to be conducted in this cohort of participants. Secondly, this trial included only a small number of participants, whereas a larger sample size would have helped to quantify the changes resulting from this exercise training more accurately. Furthermore, the observed adaptations are limited to the duration of our intervention; a longer intervention could have resulted in greater adaptations. It should also be considered that there are differences in the biometric variables at the beginning of the investigation, probably related to the randomization process. Another limitation is that a third training group that did not receive nutrition education was not created because we preferred that the larger sample benefit from this program. The Borg scale is a subjective and qualitative measurement tool; therefore, it is not global; intensity should also be measured with objective data. Finally, it should be noted that the CG did not perform any structured physical activity during the 14 weeks of intervention, which may have contributed to their deterioration.

Future research should measure body composition with the standard reference model; dual-energy X-ray absorptiometry (DXA). In addition, more consistent methods of analysis could be used to obtain more representative information for the study population. The variable sleep quality of the participants also should be controlled. It has previously been observed that more than 50% of people aged 65 years or older have sleep disorders. These sleep disorders are associated with decreased cognitive function, increased falls, worsened health status, and increased mortality (64). In addition, a stress test with gas analyser should be performed. Always trying to obtain a larger sample size.

## Conclusion

In conclusion, the key observation of this study is that, in addition to the known physical benefits for older populations such as strength, functional capacity and flexibility, resistance training in an aquatic environment, along with nutritional education are beneficial for improving body composition and life satisfaction. Future research should consider the frequency and duration of training in relation to psychological functioning to identify when changes occur, thus more accurately defining the duration and intensity of training needed to obtain benefits. Under supervised conditions, the intervention is safe and, based on the results, should be further investigated in a larger cohort of male and female participants.

## Data availability statement

The original contributions presented in this study are included in the article/supplementary material, further inquiries can be directed to the corresponding author.

## Ethics statement

The studies involving human participants were reviewed and approved by the Alicante University Ethical Committee. The patients/participants provided their written informed consent to participate in this study.

## Author contributions

AM-R and PM-P: conceptualization, validation, and supervision. AM-R, JG, RY-S, and PM-P: methodology. AM-R, RY-S, and JG: software. AM-R, BC-C, and RY-S: formal analysis. AM-R, BC-C, RY-S, JG, and PM-P: investigation. BC-C and PM-P: resources. BC-C, RY-S, and JG: data curation. AM-R, BC-C, and PM-P: writing—original draft preparation. PM-P: writing—review and editing. AM-R and RY-S: visualization. All authors have read and agreed to the published version of the manuscript.

## Footnotes

1
http://www.consort-statement.org

2
www.clinicaltrials.gov
